# The NFATc1/P2X7 receptor relationship in human intervertebral disc cells

**DOI:** 10.3389/fcell.2024.1368318

**Published:** 2024-04-04

**Authors:** Maria Pina Notarangelo, Letizia Penolazzi, Elisabetta Lambertini, Simonetta Falzoni, Pasquale De Bonis, Cristina Capanni, Francesco Di Virgilio, Roberta Piva

**Affiliations:** ^1^ Department of Neuroscience and Rehabilitation, University of Ferrara, Ferrara, Italy; ^2^ Department of Chemical, Pharmaceutical and Agricultural Sciences of the University of Ferrara, Ferrara, Italy; ^3^ Department of Medical Sciences, University of Ferrara, Ferrara, Italy; ^4^ Neurosurgery Department, Sant’Anna University Hospital, Ferrara, Italy; ^5^ CNR Institute of Molecular Genetics “Luigi Luca Cavalli-Sforza”, Unit of Bologna, Bologna, Italy; ^6^ IRCCS Rizzoli Orthopedic Institute, Bologna, Italy

**Keywords:** intervertebral disc cells, P2X7 purinergic receptor, NFATc1 transcription factor, hypoxia-inducible factor-1α, proximity ligation assay, lamin A/C, intervertebral disc degeneration

## Abstract

A comprehensive understanding of the molecules that play key roles in the physiological and pathological homeostasis of the human intervertebral disc (IVD) remains challenging, as does the development of new therapeutic treatments. We recently found a positive correlation between IVD degeneration (IDD) and P2X7 receptor (P2X7R) expression increases both in the cytoplasm and in the nucleus. Using immunocytochemistry, reverse transcription PCR (RT-PCR), overexpression, and chromatin immunoprecipitation, we found that NFATc1 and hypoxia-inducible factor-1α (HIF-1α) are critical regulators of P2X7R. Both transcription factors are recruited at the promoter of the *P2RX7* gene and involved in its positive and negative regulation, respectively. Furthermore, using the proximity ligation assay, we revealed that P2X7R and NFATc1 form a molecular complex and that P2X7R is closely associated with lamin A/C, a major component of the nuclear lamina. Collectively, our study identifies, for the first time, P2X7R and NFATc1 as markers of IVD degeneration and demonstrates that both NFATc1 and lamin A/C are interaction partners of P2X7R.

## 1 Introduction

Mobility of the intervertebral joints is ensured by very complex fibrocartilaginous anatomical structures, the intervertebral discs (IVDs) (Raj, 2008). Degeneration of discal tissue (intervertebral disc degeneration, IDD) due to physiological aging, injury, or trauma is a spinal disorder that affects approximately 80% of the population worldwide but, to date, remains without a cure (Wen et al., 2022; Samanta et al., 2023). Research on this area is largely aimed at developing effective targeted therapies relying on promising data from in-depth investigations of molecular alterations observed in the degenerated IVD microenvironment. Molecules associated with inflammation, oxidative stress, senescence, and degradation of the extracellular matrix are at the center of interest of numerous studies aimed at understanding the different phenomena that support the onset of IDD (Kamali et al., 2021; Zhang et al., 2022). More recently, interest has also turned to molecular factors that may play critical roles in regulating discogenic differentiation, such as specific transcription factors or effectors of epigenetic modifications whose activity is conditioned by the complex interaction of IVD cells with the microenvironment (Lin et al., 2019; Ghaffarpasand et al., 2023; Kang et al., 2023).

The main objective of this study was to investigate the possible roles of proteins so far not closely associated with the pathophysiology of the IVD. Starting evidence was that during daily activities, the human spine is exposed to mechanical forces that promote the release and accumulation of ATP in the extracellular space of the IVD microenvironment (Gonzales et al., 2015; Zimmermann, 2016; Fearing et al., 2018; Yin et al., 2023). This event heavily affects IVD cell metabolism and responses to mechanical stress. In fact, continuous exposure to mechanical stimuli elicits an adaptive response characterized by differential expression of specific genes and changes in extracellular matrix (ECM) production (Gonzales et al., 2015). We are referring both to a physiological context where ATP, once outside the cell, acts as an autocrine/paracrine signal, and to a damaged context in which the extracellular ATP released by damaged or stressed IVD cells acts as a damage-associated molecular pattern (DAMP) signal (Setton and Chen, 2006; Gonzales et al., 2015; Zimmermann, 2016). Accordingly, IVD cells constantly experience substantial levels of ATP in the extracellular microenvironment, both to meet physiological needs and as a consequence of IDD-associated degeneration/inflammation. It is therefore reasonable that IVD cells are equipped with several ATP-sensitive receptors as well as ATP-degrading enzymes that may influence each other to propagate or terminate the physiological or pathological signal in a concerted way. However, these systems have not been extensively studied in IVDs. Among the relevant molecules, there are NFATc1 and the P2X7 receptor (P2X7R), which are the focus of the current investigation.

NFATc1, a transcription factor belonging to the nuclear factor of activated T-cell family, was originally identified in T cells as an inducer of cytokine gene expression but was then shown to play a much broader role in various pathophysiological processes, including osteoclastogenesis, angiogenesis, chondrogenesis, and adipogenesis (Macian, 2005). Activation of NFATc1 usually requires translocation from cytosol to the nucleus and is controlled by intracellular calcium levels and the calmodulin-dependent phosphatase, calcineurin (Crabtree and Olson, 2002). Regarding the skeletal system, while there is evidence demonstrating a role of NFATc1 in bone and cartilage cell homeostasis (Winslow et al., 2006; Negishi-Koga and Takayanagi, 2009), NFATc1-mediated signaling has indeed received little attention in IVD tissue, except a recent paper focusing on the role of the calcineurin/NFATc1 pathway in hypoxic adaption of IVD cells (Huang et al., 2020).

P2X7R is an ATP-gated plasma membrane ion channel allowing an influx of Ca^2+^ and Na^+^ and an efflux of K^+^ (Adinolfi et al., 2005; Di Virgilio et al., 2018). P2X7R can either promote cell survival and proliferation by acting as a mediator of purinergic signaling in an autocrine/paracrine way or trigger cell death by acting as a cytotoxic receptor (Burnstock, 2006; Lara et al., 2020). In this highly versatile function, P2X7R plays an established role in several cellular pathophysiological musculoskeletal responses, including regulation of bone and cartilage metabolism and participation in IDD, as we recently demonstrated (Li et al., 2005; Li et al., 2021a; Penolazzi et al., 2022).

We hypothesized that NFATc1 and P2X7R might closely interact in the modulation of IVD responses. However, to date, evidence regarding the coupling between NFATc1- and P2X7R-mediated signaling pathways is poor and mostly from cell lines (Adinolfi et al., 2009). The most convincing examples of NFATc1/P2X7R interaction derive from the investigation of P2X7-dependent proliferation of T cells or non-lymphocytic cells such as osteoblasts, microglia, and several tumor cell lines (Prasai et al., 2011; Kopp et al., 2019; Lara et al., 2020). Notably, the ability of the P2X7R to mediate the effects of high concentrations of ATP on the transcriptional activity of NFATc1 in mouse osteoblast-like cells is worth mentioning (Grol et al., 2013), as well as the activation of astrocyte NFATc1 in response to injury-triggered ATP release and activation of metabotropic purinergic receptors (Liu et al., 2023). Finally, we also recently showed that in SaOS-2 osteoblastic-like cells, NFATc1 is recruited at the P2X7R gene promoter, thereby triggering P2X7R upregulation (Bergamin et al., 2021).

Moving from these considerations, the present study was designed in particular to clarify the NFATc1/P2X7R relationship, evaluate the role of NFATc1 in the regulation of the *P2RX7* gene, and analyze the participation of P2X7R in protein complexes potentially implicated in signaling pathways in the IVD microenvironment.

## 2 Materials and methods

### 2.1 IVD tissue collection and cell isolation

This study was approved by the Ethics Committee of the University of Ferrara and University S. Anna Hospital (Ferrara, Italy) (protocol no. 160998; approved 17 November 2016), and written informed consent was obtained from each patient (in full accordance with the Declaration of Helsinki). Human lumbar IVD tissues were collected as surgical waste from 42 patients who were undergoing surgical discectomy (Pfirrmann grades I/V, mean age of 55 years, 25 male and 17 female patients). Patients with tumor infiltration, diabetes mellitus, spondylolisthesis, serious systemic disease, ankylosing spondylitis, and HIV, HBV, and HCV infections were excluded (see Table 1 for patient information and Table 2 for experimental details for each sample). Nucleus pulposus tissue from each sample was macroscopically dissected from the annulus fibrosus and was subjected to enzymatic digestion using 1 mg/mL type IV collagenase for 5 h at 37°C in DMEM HG/Ham’s F12. Once the digestion was terminated, the cell suspension was filtered through a Falcon™ 70 µm nylon cell strainer (BD Biosciences, Franklin Lakes, NJ, United States). Subsequently, the cells were centrifuged at 300 x g for 10 min at room temperature (RT), and the supernatant was discarded. The cells were resuspended in a basal medium [high-glucose Dulbecco’s modified Eagle’s medium (DMEM HG/F12) containing 10% fetal calf serum (FCS), 100 mg/mL of streptomycin, 100 U/mL of penicillin, and 1% glutamine] and seeded in polystyrene culture plates at a density of 10,000 cells/cm^2^ and subcultured up to passage 3 (Penolazzi et al., 2018). Due to the small biopsy size and low proliferation rate of primary IVD cells in a monolayer, a single donor could not be used for all required experiments. Therefore, different donors were randomly assigned to the specific experiments.

**TABLE 1:** Human IVD sample information. IVD, intervertebral disc; M, male; F, female.

*Donor*	*Age*	*Sex*	*IVD level*	*Duration of symptoms prior to surgery*	*Pfirrmann grade*
*1*	34	M	L5–S1	2 months	I
*2*	51	M	L4–L5	2 months	I
*3*	56	M	L3–L4	3 months	I
*4*	38	F	L5–S1	6 months	II
*5*	36	M	L3–L4	2 months	II
*6*	71	M	L4–L5	10 months	II
*7*	37	M	L4–L5	2 months	II
*8*	56	M	L4–L5	-	III
*9*	55	F	L4–L5	2 months	III
*10*	63	M	L4–L5	4 months	III
*11*	63	F	L5–S1	1 month	III
*12*	48	F	L4–L5	1 month	III
*13*	54	M	L4–L5	2 months	III
*14*	54	M	L4–L5	12 months	IV
*15*	80	M	L4–L5	11 months	IV
*16*	56	F	L4–L5	-	IV
*17*	51	M	L5–S1	6 months	IV
*18*	70	M	L4–L5	2 months	IV
*19*	54	M	L3–L4	1 month	V
*20*	31	M	L5–S1	1 month	III
*21*	52	F	L3–L4	10 months	IV
*22*	53	M	L3–L4	9 months	IV
*23*	37	F	L5–S1	3 months	III
*24*	24	M	L4–L5	4 months	III
*25*	26	F	L5–S1	12 months	IV
*26*	53	M	L3–L4	4 months	III
*27*	65	M	L2–L3	11 months	IV
*28*	79	M	L3–L4	24 months	III
*29*	65	F	L4–L5	4 months	IV
*30*	65	M	L4–L5	9 months	IV
*31*	81	F	L4–L5	Several years	IV
*32*	78	F	L2–L3	8 months	IV
*33*	52	M	L2–L3	-	III
*34*	56	F	L3–L4	3 months	III
*35*	45	M	L4–L5	7 months	III
*36*	50	M	L5–S1	8 months	IV
*37*	50	F	L5–S1	5 months	IV
*38*	56	M	L5–S1	5 months	IV
*39*	76	F	L5–S1	10 months	V
*40*	64	F	L4–L5	-	IV
*41*	80	F	L4–L5	12 months	IV
*42*	51	F	L4–L5	9 months	III

**TABLE 2:** Human IVD samples used for each type of analysis.

	*Donor*
*Immunohistochemistry*	1–19
*Immunocytochemistry*	20–25 and 36–41
*Immunofluorescence*	27, 28, and 31
*Gene expression analysis*	23–25, 37, 40, and 41
*CoIP*	29–32
*Extracellular ATP quantification*	20, 40, and 41
*In situ* *PLA*	27, 28, and 31
*Chromatin immunoprecipitation* *(ChIP) assay*	26, 27, 33–37, and 40–42

Where required, IVD cells were exposed to 200 μM CoCl_2_ for 24 h to simulate a hypoxic environment.

### 2.2 Reagents

The primary antibodies against SOX9 (# sc-20095), aggrecan (ACAN, # sc-33695), HIF-1α (H1α67, # sc-53546), NFATc1 (7A6, # sc-7294; H-10, # sc-1783; H-110, # sc-13033), lamin A/C (E-1, # sc-376248), and normal mouse IgG_1_ (# sc-3877) were purchased from Santa Cruz Biotechnology, Inc. (Dallas, TX, United States), while anti-collagen type II α1 chain (COL2a1; # ab3092) antibody was purchased from Abcam (Cambridge, United Kingdom), anti-HIF-1α (H1 alpha67, # NB100–134) was purchased from Novus Biologicals (Centennial, CO, United States), anti-P2X7R C-terminal (# APR-004) was purchased from Alomone Labs (Jerusalem, Israel), and anti-P2X7R C-terminal (#P8232) and anti-P2X7R extracellular loop (#P9122) were purchased from Sigma-Aldrich, Merck KGaA (Milan, Italy). The chicken anti-mouse IgG TRITC-conjugated (# ab6811) and sheep anti-rabbit IgG FITC-conjugated (# ab6791) secondary antibodies were purchased from Abcam; the goat anti-mouse IgG horseradish peroxidase (HRP)-conjugated (#P0447) secondary antibody was supplied by Dako, Agilent Technologies, Inc. (Santa Clara, CA, United States), while the goat anti-rabbit IgG HRP-linked (# 7074) secondary antibody was purchased from Cell Signaling Technology (Danvers, MA, United States). DMEM HG, Ham’s F12, FCS, L-glutamine, antibiotics (penicillin and streptomycin), and 1× phosphate-buffered saline (PBS) were purchased from Euroclone S.p.A (Milan, Italy). Type IV collagenase, Triton X-100, phorbol 12-myristate 13-acetate (PMA), protease inhibitor cocktail, Magna ChIP Protein A + G magnetic beads (# 16-663), Immobilon Western Chemiluminescent HRP substrate (# WBKLS0500), and Bradford reagent (#B6916) were purchased from Sigma-Aldrich, Merck KGaA. Lipofectamine 2000, ionomycin, and ProLong Gold Antifade Mountant with DNA stain DAPI (4′, 6-diamidino-2-phenylindole) (#P36935) were purchased from Thermo Fisher Scientific (Waltham, MA, United States).

### 2.3 Immunohistochemistry

Small fragments of each IVD sample were rinsed with PBS 1×, fixed in 4% buffered paraformaldehyde for 24 h at 4°C, embedded in paraffin, and cross-sectioned (5 µm thick). Histochemical sections were deparaffinized, rehydrated, and heated in sodium citrate (pH 6) for antigen retrieval. Slides were then processed with 3% H_2_O_2_ in PBS 1× for 5 min and with a blocking solution [PBS 1×/1% bovine serum albumin (BSA)/10% FCS] for 30 min at RT. For immunohistochemical evaluation, sections were incubated overnight with a primary antibody against NFATc1 (1:25 dilution, # sc-1783) at 4°C, followed by treatment with ImmPRESS-HRP universal polymer reagent (horse anti-mouse/rabbit IgG, kit # MP-7500, Vector Laboratories, Inc., Burlingame, CA, United States) for 30 min. Then, the reactions were developed using 3,3′-diaminobenzidine (DAB) solution (ImmPACT™ DAB, # SK-4105, Vector Laboratories, Inc.), and the sections were counterstained with hematoxylin and mounted in glycerol. The stainings were quantified using a computerized video camera-based image analysis system (NIH; United States ImageJ software, public domain available at http://rsb.info.nih.gov/nih-image/) under brightfield microscopy (Nikon Eclipse 50i; Nikon Corporation). For the analysis of sections, positive cells in the area were counted, and protein levels were expressed as % of positive cells (10 fields per replicate, 5 sections per sample).

### 2.4 Immunocytochemistry

Immunocytochemistry analysis was performed using the ImmPRESS^®^-HRP universal polymer kit. Cells were plated at a density of 20,000 cells/cm^2^ in 24-well plates. The cells were fixed in cold 100% methanol for 10 min at RT and permeabilized with 0.2% (v/v) Triton X-100 in Tris-buffered saline (TBS) 1× for 10 min at RT. The cells were treated with 3% H_2_O_2_ in TBS 1× for 10 min at RT and incubated in 2.5% normal horse serum (Vector Laboratories, Inc.) for 15 min at RT. After incubation in a blocking serum, primary antibodies against SOX9 (1:800), ACAN (1:200), COL2a1 (1:200), P2X7R (1:1,000; # APR-004), NFATc1 (1:500; # sc-13033), and HIF-1α (1:100; # sc-53546) were added and incubated overnight at 4°C. After rinsing in TBS 1×, the cells were incubated for 30 min at RT with ImmPRESS-HRP universal polymer reagent (horse anti-mouse/rabbit IgG) and then stained with substrate/chromogen mix (ImmPACT™ DAB). After washing, the cells were mounted in glycerol/PBS 1× (9:1) and observed under a Nikon Eclipse 50i optical microscope. Quantitative image analysis of immunostained cells was obtained using a computerized video camera-based image analysis system (with ImageJ software, http://fiji.sc/Fiji) under brightfield microscopy. In brief, images were grabbed with single stain, without carrying out nuclear counterstaining with hematoxylin, and unaltered TIFF images were digitized and converted to black and white pictures to evaluate the distribution of relative gray values (i.e., the number of pixels in the image as a function of gray value 0–256), which reflected chromogen stain intensity. Images were then segmented using a consistent arbitrary threshold of 50% to avoid a floor or ceiling effect and binarized (black *versus* white); the total black pixels per field were counted, and average values were calculated for each sample. At least 10 fields per replicate were subjected to densitometric analysis. We performed the quantification of pixels per 100 cells and not per area in order to take into account the different cell morphologies and confluences (Penolazzi et al., 2019).

### 2.5 Cell transfection

Human IVD cells were seeded in 24-well plates at a density of 40,000 cells/well. The cells were transfected with 100 or 200 ng of NFATc1/A (pRSV NFATc1/A) expression vector using Lipofectamine 2000 according to the manufacturer’s instructions. After 16 h of transfection, the medium was replaced with fresh medium, and the cells were stimulated, where required, with 20 nM PMA and 1 µM ionomycin for another 3 h. Then, the cells were harvested and analyzed for RNA levels by reverse transcription quantitative PCR (RT-qPCR) and for protein expression by immunocytochemistry.

### 2.6 RNA isolation and RT-qPCR

Total RNA was isolated from IVD cells using the RNeasy micro kit (# 74004, Qiagen GmbH, Hilden, Germany) according to the manufacturer’s instructions. A NanoDrop™ ND1000 UV-VIS spectrophotometer (Isogen Life Science B.V., Utrecht, Netherlands) was used to measure RNA quantity. cDNA was synthesized from total RNA in a 20 µL reaction volume using the High-Capacity cDNA RT kit (Thermo Fisher), according to the manufacturer’s instructions. qPCR analysis was performed using TaqMan universal master mix II (# 4440043, Thermo Fisher) and probes for human NFATc1 (assay no. Hs00542675_m1, Thermo Fisher) and *P2RX7* (assay no. Hs00175721_m1, Thermo Fisher). Experimental reactions were conducted by preincubation (95°C for 10 min) and amplification (95°C for 15 s and 60°C for 60 s) for 40 cycles. RPL13a (assay no. Hs04194366_g1, Thermo Fisher) was used for normalization of mRNA expression. Gene expression was assessed using a CFX96™ PCR detection system (Bio-Rad Laboratories, Inc., Hercules, CA, United States), and relative gene expression was calculated using the comparative 2^−ΔΔCt^ method and expressed as fold change. All reactions were performed in triplicate.

### 2.7 Immunofluorescence

Cells were seeded on glass coverslips, put into 24-well plates at a density of 20,000 cells/well, and fixed in 100% methanol for 7 min at RT. After washes, the cells were blocked with 4% BSA/PBS 1×. Afterward, the cells were incubated with the following primary antibodies: anti-NFATc1 (1:10, # sc-7294) overnight at 4°C, anti-P2X7R (1:300, #P8232) and anti-lamin A/C (1:3000, # sc-376248) 1 h at RT. Where required, P2X7R-blocking peptide (# AB5246, Merck KGaA, Darmstadt, Germany) was added to the primary antibody at a 1:1 ratio (Penolazzi et al., 2022; Penolazzi et al., 2023). Appropriate conjugated secondary antibodies were then used for 1 h at RT (chicken anti-mouse IgG, 1:200; sheep anti-rabbit IgG, 1:600). The coverslips were mounted with ProLong Gold Antifade with DNA stain DAPI, and immunofluorescence analysis was performed using a Nikon Eclipse Ni fluorescence microscope equipped with a digital CCD camera and NIS Elements AR 4.3 software.

### 2.8 Immunogold labeling and electron microscopy

Cells were fixed in 2% paraformaldehyde/PBS for 1 h, permeabilized with 0.1% Triton X-100, and blocked with PBS/2% BSA (Tao-Cheng et al., 2021). The cells were labeled overnight with anti-P2X7R (1:20 dilution, #P8232) and then incubated with Protein A—20 nm colloidal gold-labeled (#P6855, Sigma-Aldrich). Finally, the cells were fixed in glutaraldehyde 2.5% phosphate buffer and osmium tetroxide 2%, dehydrated, and araldite-embedded (Sigma-Aldrich). The ultra-thin sections of a selected area were contrasted with uranyl acetate and lead citrate and observed using a ZEISS EM910 transmission electron microscope (ZEISS, Jena, Germany). Images were captured using an Olympus Megaview III digital camera (Olympus Co., Tokyo, Japan).

### 2.9 Co-immunoprecipitation and immunoblotting

For the co-immunoprecipitation (CoIP) assay, IVD cells were lysed in ice-cold (RIPA) buffer [50 mM Tris-HCl, pH 7.8, 1% NP-40, 150 mM NaCl, 0.5% SDC (sodium deoxycholate), and 0.1% SDS (sodium dodecyl sulfate)] supplemented with protease inhibitors for 15 min at 4°C. The cells were centrifuged at 12,000 x g for 10 min at 4°C to remove cell debris. The Bradford method was used for protein quantification. Five percent of the cell lysates were kept as the input sample, and 1 mg of protein lysates was incubated with 10 μg NFATc1 (# sc-7294) antibody for 12 h at 4°C, followed by incubation with 20 µL of Magna ChIP Protein A + G magnetic beads for 2 h at 4°C with gentle rocking. Normal mouse IgG was used as a negative control. The beads were collected by centrifugation at 6,000 rpm for 1 min at 4°C and washed four times with RIPA buffer and once with PBS 1×. The immunocomplexes were released by heating the beads in 15 μL of Laemmli sample buffer. The proteins were then separated on a 10% SDS-PAGE gel and transferred onto an Immobilon-P PVDF membrane. Then, the membrane was blocked with 5% (w/v) NFDM (non-fat dry milk) in TBS-0.1% Tween-20 (TBST) for 1 h at RT and incubated with primary antibodies against NFATc1 (1:250, # sc-1783) and P2X7R (1:300; #P9122) for 16 h at 4°C. After washes, the membrane was incubated with appropriate secondary antibodies (1:2000 goat anti-mouse HRP-conjugated; 1:3000 goat anti-rabbit HRP-conjugated) for 1 h at RT, followed by detection using an enhanced chemiluminescence method (Immobilon Western Chemiluminescent HRP substrate).

### 2.10 *In situ* proximity ligation assay

*In situ* proximity ligation assay (PLA) was performed using Duolink^®^ *In Situ* Orange Starter Kit Mouse/Rabbit (# DUO92102, Sigma-Aldrich, Merck KGaA). Cells were seeded on glass coverslips, put into 24-well plates at a density of 20,000 cells/well, and fixed in 100% methanol for 7 min at RT. After washes, the cells were blocked with 4% BSA/PBS 1× and incubated with the following primary antibodies: anti-NFATc1 (1:10, # sc-7294) overnight at 4°C, anti-P2X7R (1:300, #P8232) and anti-lamin A/C (1:3000, # sc-376248) 1 h at RT. Thereafter, the cells were incubated for 1 h at 37°C with secondary probes diluted to a final concentration of 1:5. Ligation solution was added to each sample and incubated in a humidity chamber for 30 min at 37°C. Later, the ligation solution was removed with wash buffer A, and an amplification solution was added and incubated in a humidity chamber for 100 min at 37°C. After washes, Duolink *in situ* mounting medium with DAPI was added, and the samples were observed using a Nikon Eclipse Ni fluorescence microscope equipped with a digital CCD camera and NIS Elements AR 4.3 software. Quantitative analysis of the PLA results was performed using Duolink ImageTool software (Sigma-Aldrich) by counting 100 cells or nuclei per sample.

### 2.11 Bioinformatic analysis

NFATc1- and HIF-1α-binding sites within the *P2RX7* promoter region (−2000/+1) were predicted by the JASPAR Core database (http://jaspar.genereg.net) with a relative score threshold of 85%.

### 2.12 Chromatin immunoprecipitation assay

Chromatin immunoprecipitation (ChIP) assays were performed using the ChIP assay kit and protocol provided by Millipore, as previously described (Penolazzi et al., 2019). IVD cells were serum-starved for 16 h in DMEM HG/F12. The cells were cross-linked with 1% formaldehyde in a medium for 10 min at 37°C. After cross-linking, the cells were washed with PBS 1× twice and resuspended in cell SDS lysis buffer (50 mM Tris-HCl (pH 8.0), 10 mM EDTA, 1% SDS, and protease inhibitor mixture) for 15 min on ice. The lysates were subjected to sonication to obtain 200–500-bp fragments of DNA (10 min cycle, 10 s pulses; 40% amplitude) using a “Vibra-Cell” VC 130 (Sonics, Newtown, CT, United States). Sonicated chromatin was cleared by centrifugation at 13,000 rpm for 10 min and then diluted 10-fold with dilution buffer [20 mM Tris-HCl (pH 8.0), 1.2 mM EDTA, 0.01% SDS, 1.1% Triton X-100, 167 mM NaCl, and protease inhibitor mixture]. Protein/DNA complexes were incubated on a rotating platform overnight at 4°C using 5 µg of anti-NFATc1 (# sc-7294), or anti-HIF-1α (# NB100–134), or normal mouse IgG_1_ and 20 µL of Magna ChIP Protein A + G magnetic beads. Immunoprecipitated chromatin complexes were sequentially washed (four times each) with the following buffers: low-salt wash buffer, high-salt wash buffer, LiCl wash buffer, and TE buffer. All wash procedures were performed with a magnetic separator. Precipitated complexes were eluted from the beads in fresh elution buffer (1% SDS, 0.1 M NaHCO_3_) for 30 min at RT and subsequently heated at 65°C for 5 h to reverse cross-links. Samples were then digested with proteinase K for 1 h at 45°C, and DNA was purified using the Wizard(R) SV Gel and PCR Clean-Up System kit (# A9281, Promega, Madison, WI, United States), according to the manufacturer’s instructions. Quantitative real-time PCR was performed with CFX96 real-time detection system (Bio-Rad) using iTaq Universal SYBR Green SuperMix (Bio-Rad). The Input fractions (1% of the cell lysate before immunoprecipitation) were used as the internal positive control. ChIP-qPCR data were calculated as a fold enrichment using the 2^−ΔΔCt^ method and normalized against the IgG signal.

### 2.13 *In vitro* measure of ATP levels

ATP was measured with the ENLITEN rLuciferase/Luciferin reagent (# FF 2021, Promega) with the IVIS Lumina luminometer (PerkinElmer, Hopkinton, MA, United States) in the supernatant of IVD cells. In brief, to measure eATP, cells were plated at a density of 50,000 cells/well in 24-well plates. Then, the culture medium was discarded immediately before the measurement and replaced with 450 µL of fresh medium (without serum) supplemented with 50 µL of rLuciferase/Luciferin reagent for each well. The total photon flux was normalized to the cell protein content (µg).

### 2.14 Statistical analysis

The data are expressed as the mean ± standard deviation. GraphPad Prism 8.0 software (GraphPad Software, Inc.) was used for all statistical analyses. Data were compared using a one-way ANOVA, followed by Tukey’s *post hoc* test for multiple comparisons. Each experiment was repeated at least three times. An unpaired Student’s *t*-test was used to compare differences between two groups. p< 0.05 was considered statistically significant.

## 3 Results

### 3.1 Expression of P2X7R and NFATc1 in IVD cells

**FIGURE 1:**
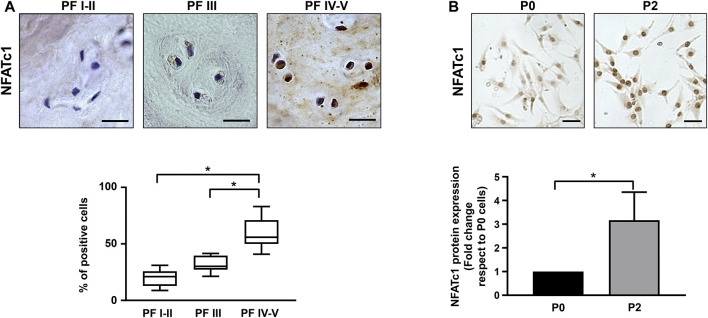
NFATc1 expression in IVD. **(A)** Immunohistochemical analysis of NFATc1 was performed on IVD tissues at different Pfirrmann (PF) grades. Quantification was reported and expressed as % of positive cells per area (3–5 sections per sample; PF I–II group, n = 7; PF III group, n = 6; PF IV–V group, n = 6). *p < 0.001 PF IV–V group vs PF I–II group and PF IV–V group vs PF III. **(B)** NFATc1 expression was analyzed in IVD cells during the de-differentiation process from P0 to P2 passages by immunocytochemistry, and representative photomicrographs are reported. Protein levels were quantified by densitometric analysis of immunocytochemical pictures using ImageJ software. Quantitative analysis of at least 10 fields per replicate was performed. Protein levels are expressed as fold change relative to P0 cells. Data are presented as the mean ± SD (n = 4). *p < 0.05. Scale bar = 20 μm.

IVD biopsies with different levels of degeneration and classified according to the Pfirrmann grading system were analyzed by immunohistochemistry for NFATc1 expression. As reported in Figure 1A, the percentage of NFATc1-positive cells were significantly higher in IVD samples showing a higher level of degeneration, suggesting that NFATc1 expression correlates with disc degeneration.

An aliquot of the IVD samples was used to set up primary cultures for the following experiments. As previously described (Penolazzi et al., 2018; Penolazzi et al., 2020) and as reported in Supplementary Figure S1A, IVD cells at passage P2 become de-differentiated and lose their chondrocyte-like phenotype, as revealed by decreased expression of typical chondrogenic markers, including aggrecan, collagen type II, and the SOX9 transcription factor (Supplementary Figure S1B). In accordance with immunohistochemical analysis, a statistically significant increase in the expression of NFATc1 was observed in IVD cells (Figure 1B). Likewise, as already shown in Penolazzi et al. (2022), expression of P2X7R increased during IVD cell *in vitro* culture at an increasing number of passages (Supplementary Figure S1B). Overall, these data demonstrate that changes in the expression of critical genes, such as those analyzed here, were found both in IVD cells after two passages in culture and in degenerated IVD tissue, strengthening the evidence that *in vitro* cellular de-differentiation recapitulates the process of degeneration.

Finally, continuing the cellular characterization, the soluble luciferase/luciferin assay revealed the presence of physiological levels of extracellular ATP (corresponding to approximately 15 nM) in the supernatant of cultured IVD cells (Supplementary Figure S1C).

**FIGURE 2:**
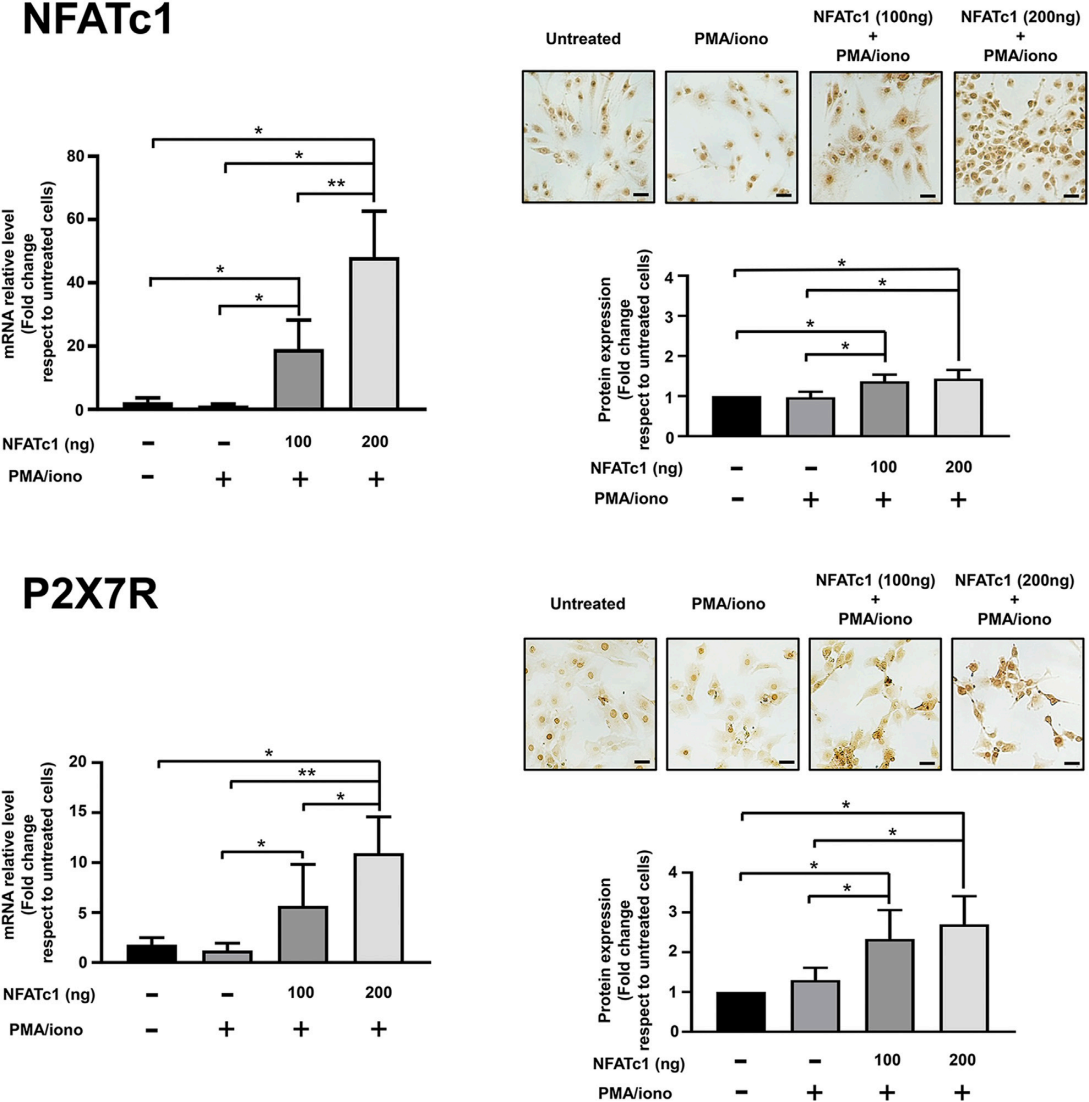
Overexpression of NFATc1 upregulates P2X7R expression in human IVD cells. IVD cells were transfected with 100 ng or 200 ng of pRSV-NFATc1/A expression vector (NFATc1) for 16 h and treated with PMA (20 nM) and ionomycin (1 μM) for 3 h. Cells were collected and subjected to RNA and protein analysis. mRNA levels of NFATc1 and P2X7R were determined by real-time RT-PCR. RNA relative expression levels were normalized to untreated cells and expressed as fold change. Data represent means ± SD (n = 3). *p < 0.01; **p < 0.0001. Protein levels of NFATc1 and P2X7R were analyzed by immunocytochemistry, and representative optical photomicrographs of immunostaining are shown. Protein levels were quantified by densitometric analysis of immunocytochemical pictures using ImageJ software. Quantitative analysis of at least 10 fields per replicate was performed. Protein levels are expressed as fold change relative to untreated cells. Data are presented as the mean ± SD (n = 3). *p < 0.05. Scale bar = 20 μm.

Clear evidence of a physiologically relevant link between NFATc1 and P2X7R stemmed from the observation that overexpression of NFATc1 upregulates P2X7R expression. As reported in Figure 2, when IVD cells were transfected with the pRSV-NFATc1/A expression vector in the presence of ionomycin and PMA to stimulate NFATc1 activity, a significant increase in P2X7R expression was found at both the mRNA and protein levels.

### 3.2 Association between P2X7R and NFATc1

**FIGURE 3:**
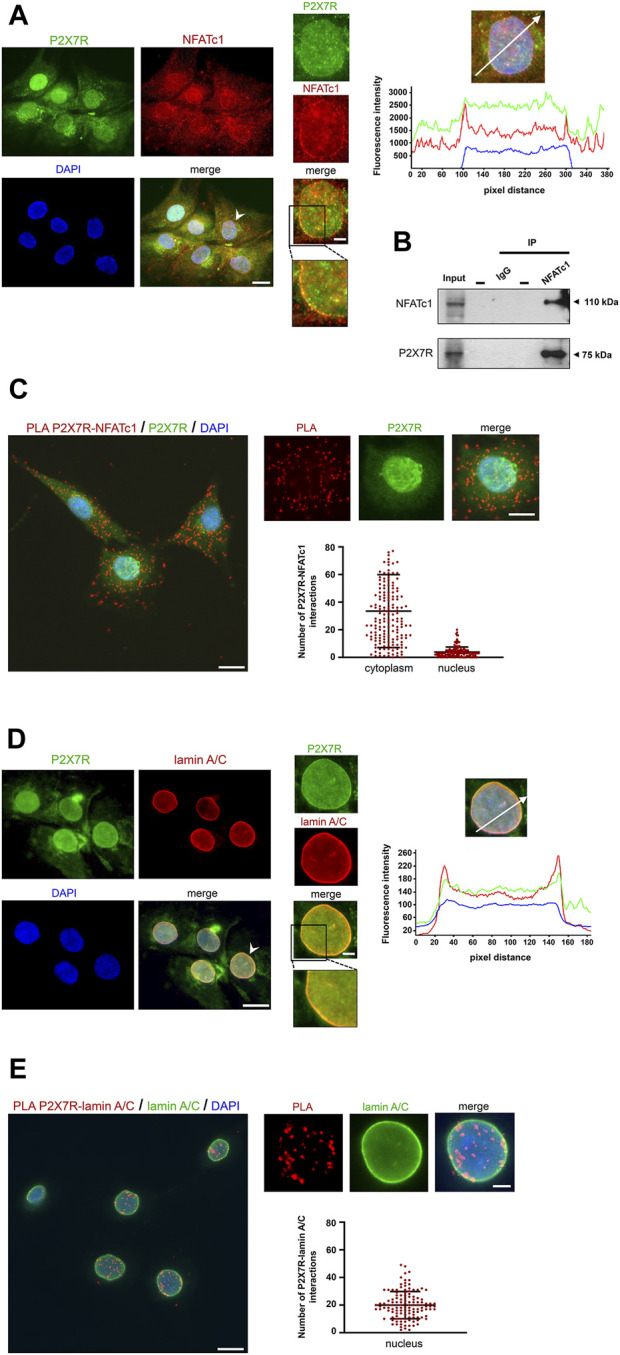
Association between P2X7R and NFATc1. **(A)** Immunofluorescence detection of P2X7R and NFATc1. The IVD cells were co-labeled with anti-P2X7R C-terminal antibody (green) and anti-NFATc1 antibody (red). Nuclei were counterstained with DAPI (blue). Representative images together with high-magnification pictures are reported (the nucleus chosen for magnification is indicated with a white arrowhead). Merge images represent an overlay of the two channels where colocalization is indicated by a color change (yellow). The graph indicates the fluorescence intensity profile along the white arrow. The blue, red, and green lines correspond to signals of DNA, NFATc1, and P2X7R, respectively. Scale bar = 10 μm; high-magnification pictures, scale bar = 2 μm. **(B)** CoIP assay to evaluate the interaction between NFATc1 and P2X7R. Immunoprecipitations of NFATc1 followed by immunoblot analyses with anti-NFATc1 and anti-P2X7R antibodies were performed in IVD cells derived from four patients with the same grade of degeneration pooled together. The negative control of immunoprecipitation (normal mouse IgG) was also included. **(C)** PLA of P2X7R and NFATc1 interactions performed in IVD cells using primary antibody against P2X7R and NFATc1. Detection of P2X7R was performed by immunofluorescence (green). Interactions between P2X7R and NFATc1 are revealed as red dots. Nuclei were counterstained with DAPI (blue). Quantification of positive PLA signals per cell is reported as a dot plot with mean (±SD, n = 3). PLA dot counts per cytoplasm and nucleus are reported separately. Data analyzed were based on an average of 100 cells. Scale bar = 10 μm; high-magnification pictures, scale bar = 5 μm. **(D)** Immunofluorescence detection of P2X7R and lamin A/C. The IVD cells were co-labeled with anti-P2X7R C-terminal antibody (green) and anti-lamin A/C antibody (red). Nuclei were counterstained with DAPI (blue). Representative images together with high-magnification pictures are reported (the nucleus chosen for magnification is indicated with a white arrowhead). Merge images represent an overlay of the two channels where colocalization is indicated by a color change (yellow). The graph indicates the fluorescence intensity profile along the white arrow. The blue, red, and green lines correspond to signals of DNA, lamin A/C, and P2X7R, respectively. Scale bar = 10 μm; high-magnification pictures, scale bar = 2 μm. **(E)** PLA of P2X7R and lamin A/C interactions performed in IVD cells using a primary antibody against P2X7R and lamin A/C. Detection of lamin A/C was performed by immunofluorescence (green). Interactions between P2X7R and lamin A/C are revealed as red dots. Nuclei were counterstained with DAPI (blue). Quantification of positive PLA signals per nucleus is reported as dot plot with mean (±SD, n = 3). Data analyzed were based on an average of 100 cells. Scale bar = 10 μm; high-magnification pictures, scale bar = 3 μm.

In a previous investigation aimed at identifying the subcellular localization of P2X7R in IVD cells, we found substantial immunoreactivity not only at canonical sites, i.e., the plasma membrane and cytoplasm, but also in the nucleus (Penolazzi et al., 2023). Control experiments demonstrating the specificity of the antibody used (Supplementary Figure S2) confirmed these data, and immunofluorescence experiments showed the localization of P2X7R and NFATc1 in both the cytoplasm and nucleus (Figure 3A). We then investigated the possible interaction between these two proteins or their assembly in a protein complex. A CoIP experiment (Figure 3B) showed that there is indeed an association between P2X7R and NFATc1, likely in the same protein complex. The subcellular localization of this association was then investigated by PLA. Unlike CoIP, which occurs in solution, PLA is performed after fixation and allows to capture transient events and also preserves low-affinity interactions of endogenously expressed proteins. Analysis of PLA images showing interaction sites indicates that the P2X7R and NFATc1 are in close proximity prevalently in the cytoplasm and less in the nucleus (Figure 3C). This evidence strongly supports our previous suggestion that IVD cells, due to the high expression level of both proteins, are an ideal model for the investigation of P2X7R/NFATc1 relationship and P2X7R localization. Particularly intriguing is the nuclear localization of P2X7R, which has been undeservedly underestimated until now. To explore this aspect further, we compared the localization of P2X7R and lamin A/C, an important component of the nuclear lamina playing many roles in healthy and diseased cells, including maintenance of structural stability, cell motility, mechanosensing, chromosome organization, gene regulation, cell differentiation, DNA damage repair, and telomere protection (Lattanzi et al., 2012; Kim et al., 2023). We found, for the first time, that P2X7R is not only localized in the nuclear matrix but also at the nuclear lamina and co-localized with lamin A/C, as revealed by the immunofluorescence experiments shown in Figure 3D. Accordingly, PLA evaluation revealed that the P2X7R is closely associated with lamin A/C (Figure 3E). This raises new intriguing questions on new and previously unanticipated roles of P2X7R.

### 3.3 Recruitment of NFATc1 and HIF-1α at the *P2RX7* gene promoter

**FIGURE 4:**
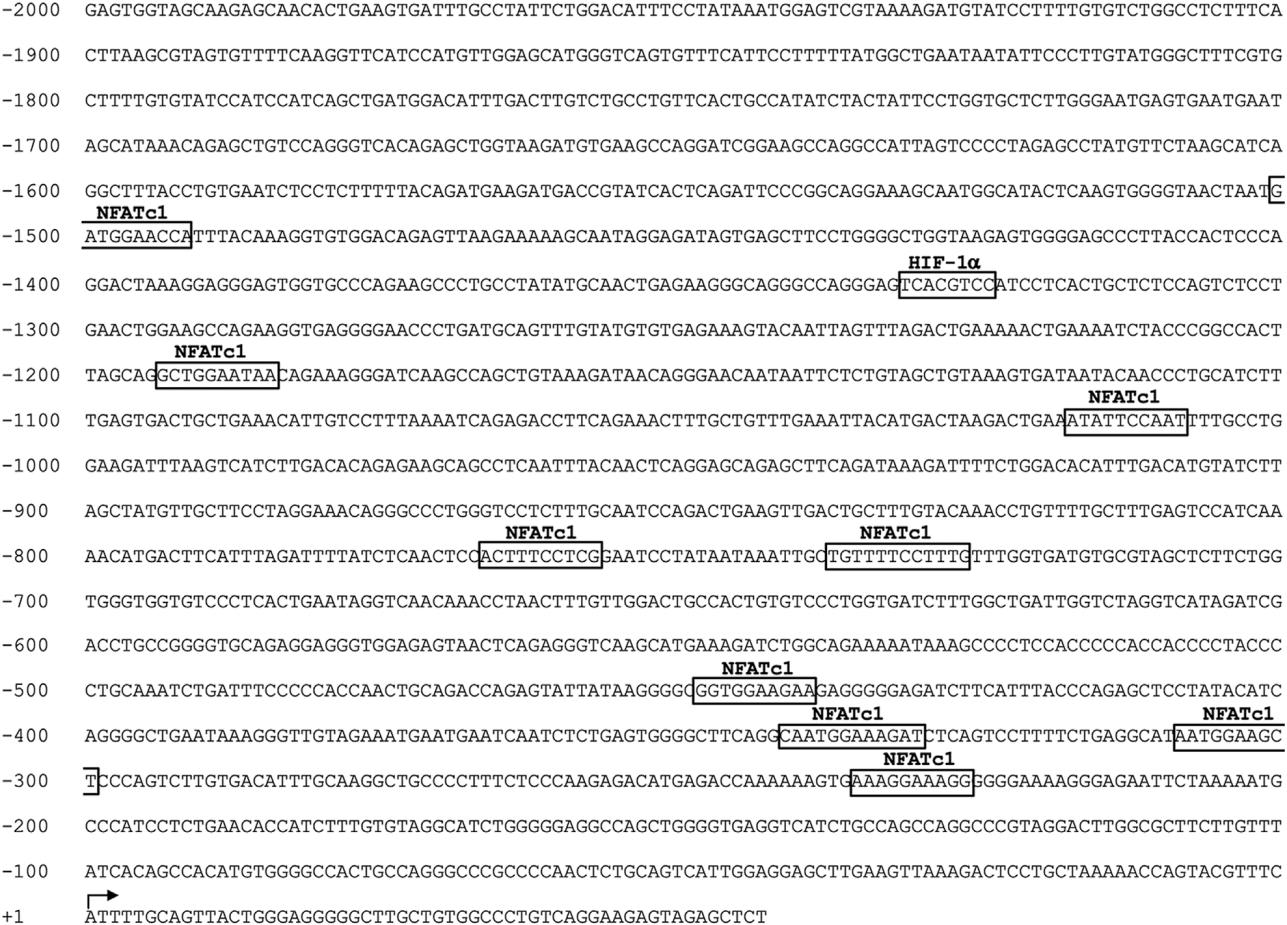
*In silico* analysis of the human *P2RX7* gene promoter region (−2000 to +32 bp). Nucleotide numbering is relative to the first nucleotide (adenine +1) of the TSS, which is indicated with an arrow. The positions of putative transcription factor-binding motifs of NFATc1 and HIF-1α identified using the JASPAR database software are boxed.

**FIGURE 5:**
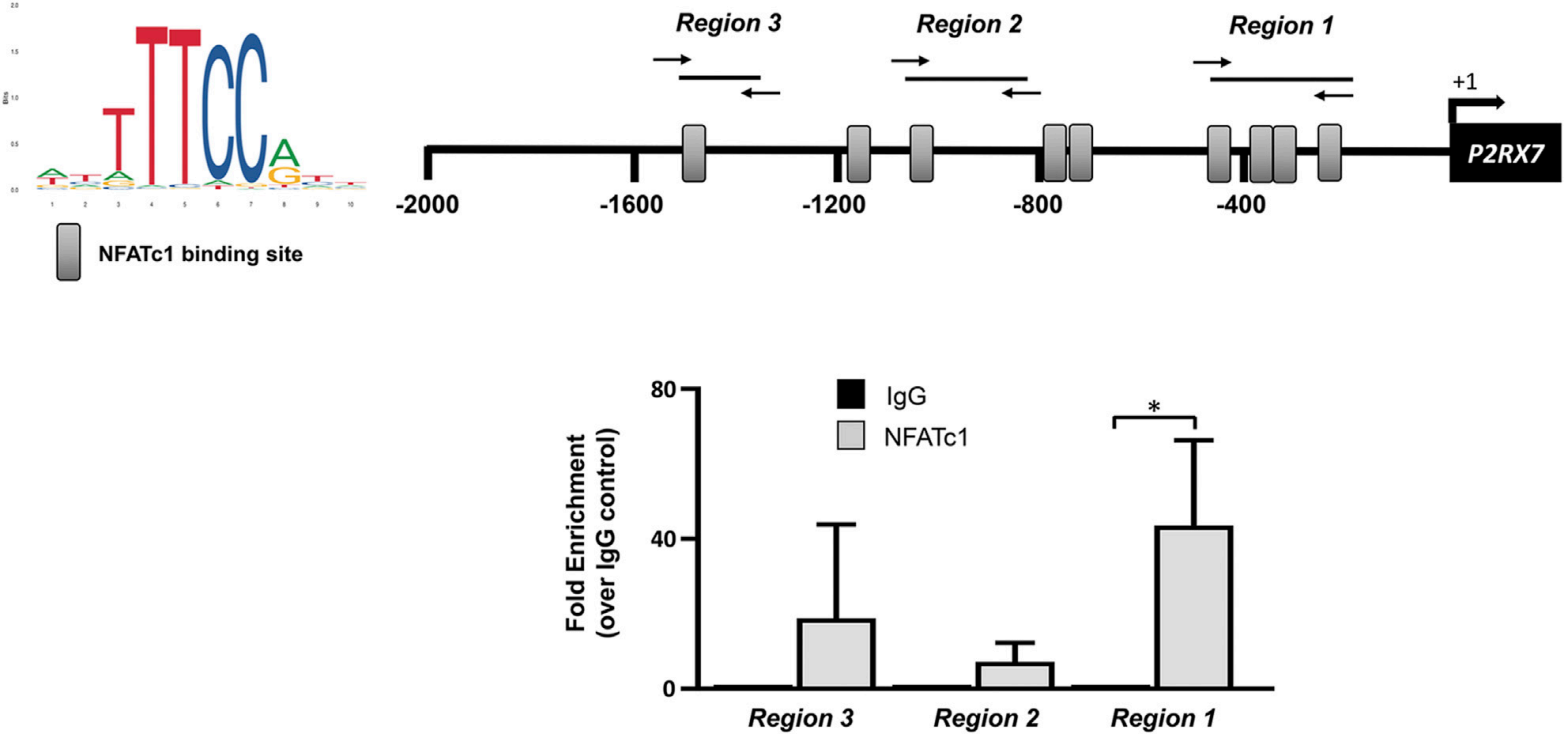
NFATc1 directly binds to the *P2RX7* promoter regions. The recognition motifs of NFATc1 from the JASPAR database and the positioning of NFATc1 potential binding sites within the *P2RX7* (−2000/+32) promoter region are reported (gray rectangles). The positions of specific primers used for qPCR amplification are also reported. The results of ChIP-qPCR were analyzed using the 2^−ΔΔCT^ method, normalized for the input signal, and presented as fold enrichment with respect to the IgG negative control. Data are expressed as the mean ± SD (n = 5). *p < 0.05.

The *P2RX7* gene promoter sequence at −2000 from the transcription start site contains nine potential binding motifs for NFAT (Figure 4). Previous ChIP experiments performed in SaOS-2 osteoblast-like cells revealed the recruitment of NFATc1 within the *P2RX7* promoter predominantly in the most proximal region (Bergamin et al., 2021). In the present study, we show that this also occurs in IVD cells, specifically in 5 of 10 samples analyzed (Figure 5). Thus, we confirmed our previous observation by showing that *P2RX7* is a direct transcriptional target of NFATc1 and that the P2X7R couples to the Ca^2+^–NFATc1 pathway (Kopp et al., 2019; Bergamin et al., 2021). Considering that, in this case, the cells were not treated with ionomycin and PMA to force translocation/activation of NFATc1 into the nucleus, these data represent the original conditions experienced by cells derived from individual patients.

**FIGURE 6:**
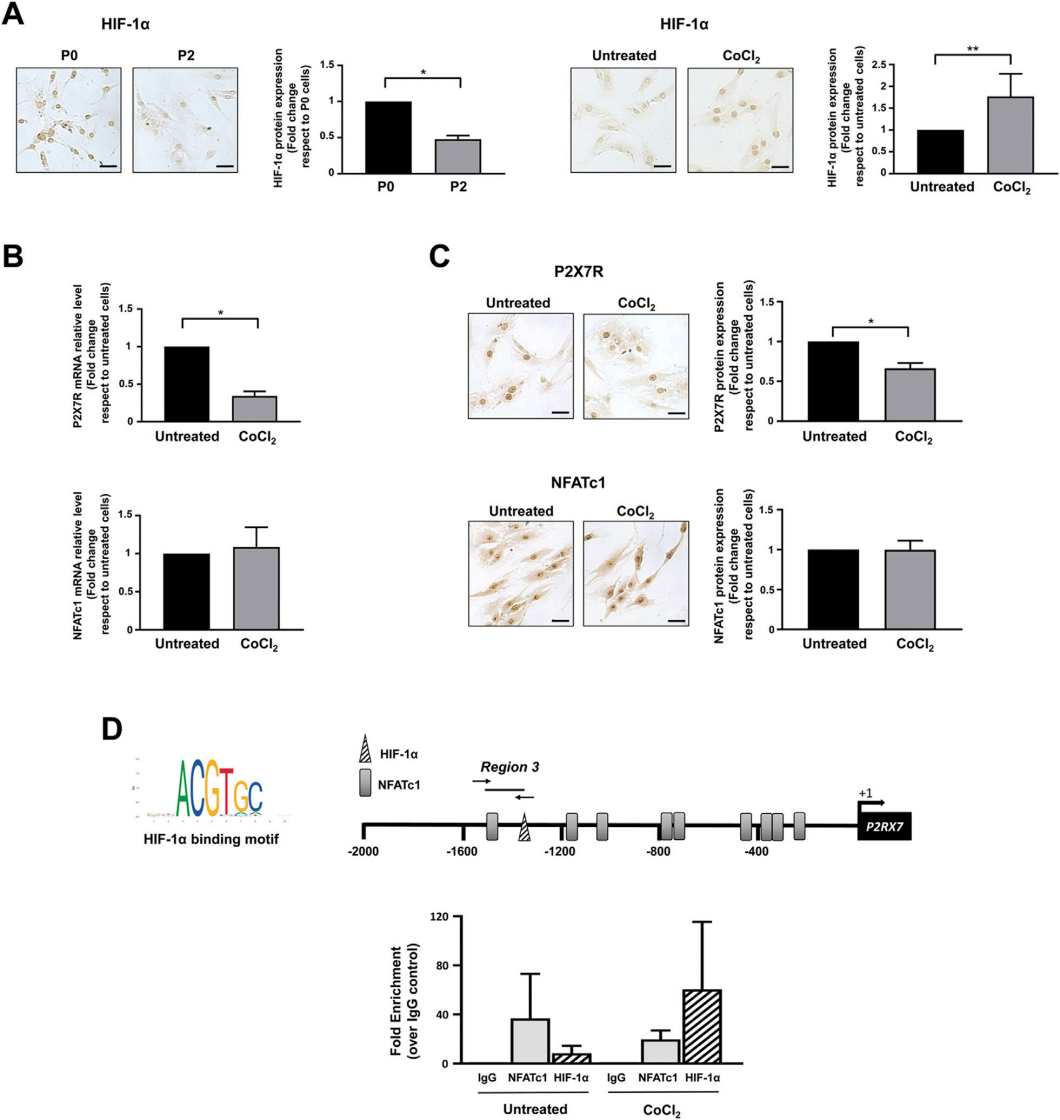
Regulation of NFATc1 and P2X7R expression under hypoxia. **(A)** HIF-1α expression was analyzed by immunocytochemistry in IVD cells during the de-differentiation process from P0 to P2 and in IVD cells cultured in the absence (untreated) or presence of CoCl_2_ for 24 h. Representative photomicrographs are reported. Protein levels were quantified by densitometric analysis of immunocytochemical pictures using ImageJ software. Quantitative analysis of at least 10 fields per replicate was performed. Protein levels are expressed as fold change relative to P0 cells or untreated cells. Data are presented as the mean ± SD (n = 4). *p < 0.0001 P2 vs P0; **p < 0.05 CoCl_2_ vs untreated. Scale bar = 20 μm. **(B, C)** IVD cells were cultured in the absence (untreated) or presence of CoCl_2_ for 24 h. Cells were then collected and subjected to RNA **(B)** and protein **(C)** analyses. mRNA levels of P2X7R and NFATc1 were determined by real-time RT-PCR. RNA relative expression levels were normalized to untreated cells and expressed as fold change. Data represent means ± SD (n = 3). *p < 0.0001 CoCl_2_ vs untreated. Protein levels were analyzed by immunocytochemistry, and representative optical photomicrographs of immunostaining are shown. Protein levels were quantified by densitometric analysis of immunocytochemical pictures using ImageJ software. Quantitative analysis of at least 10 fields per replicate was performed. Protein levels are expressed as fold change relative to untreated cells. Data are presented as the mean ± SD (n = 3). *p < 0.0001. Scale bar = 20 μm. **(D)** ChIP analysis was performed in IVD cells cultured in the absence (untreated) or presence of CoCl_2_. The recognition motifs of HIF-1α from the JASPAR database and the positioning of potential hypoxia-responsive elements (HREs) within the *P2RX7* (−2000/+32) promoter region are reported (HREs, triangle; NFATc1 potential binding sites, gray rectangles). The positions of specific primers used for qPCR amplification are also reported. Results of qPCR were analyzed using the 2^−ΔΔCt^ method, normalized for the input signal, and presented as fold enrichment with respect to the IgG negative control. Data are expressed as the mean ± SD (n = 2).

Bioinformatic analysis also revealed the presence of one HIF-1α potential binding site (−1,332/−1,325), a finding of substantial pathophysiological relevance. In fact, it is well known that healthy IVD cells experience a strong hypoxic environment and that their metabolism is under the control of HIF-1α (Semenza, 2000; Silagi et al., 2021). This transcription factor plays an important role in IVD homeostatic functions by promoting ECM synthesis, energy metabolism, and cellular adaptation to stress (Kim et al., 2021a; Li et al., 2021b; Silagi et al., 2021). Consistent with this observation, de-differentiated IVD cells in culture are characterized by a significant decrease in the level of HIF-1α (Figure 6A). Based on our previous findings showing an increase in P2X7R in the degenerated disc (Penolazzi et al., 2022), we explored the possible contribution of this transcription factor to the regulation of P2X7R expression. Interestingly, when cells are exposed to a CoCl_2_-simulated hypoxic environment (Muñoz-Sánchez and Chánez-Cárdenas, 2019) resulting in increased stability/expression of HIF-1α (Figure 6A), the expression of *P2X7R* significantly decreases (Figure 6B), while the expression of NFATc1 is not substantially affected (Figure 6C). This prompted us to investigate the recruitment of HIF-1α to the promoter of the *P2RX7* gene. ChIP experiments demonstrated that HIF-1α is recruited to the *P2RX7* promoter in 2 of 5 samples positive for NFATc1 recruitment and that HIF-1α occupancy significantly increased in CoCl_2_-treated cells (Figure 6D). Interestingly, the recruitment of NFATc1 to the *P2RX7* promoter was markedly impaired after HIF-1α recruitment promoted by CoCl_2_ treatment (Figure 6D).

## 4 Discussion

The IVD tissue microenvironment is continually adapting to changes from embryogenesis to degeneration under the regulation of numerous factors (Colombier et al., 2014). Stromal and infiltrating inflammatory cells are exposed to a plethora of extracellular stimuli which mediate specific short- and long-term cellular responses. Anabolic and catabolic factors acting on IVD cells generated within this inflammatory microenvironment (hormones, growth factors, TGF-β, ATP, cytokines, enzymes, calcium, O_2_, extracellular matrix components, etc.) have been extensively investigated, and the mode of action of many of them has been clarified (Dou et al., 2021).

What still remains to be resolved are the complicated interactions between different signaling pathways within IVD cells to unveil the mechanism through which different changes mold the adaptation to harmful stimuli, heavy physical constraints, infections, tissue injury, and the unavoidable decay due to aging/senescence. Elucidating the relationship between different molecular pathways during these adaptive changes is a major challenge. This is indeed extremely critical, in particular, for the development of therapies aimed at restoring IVD functions by correcting/targeting the factors responsible for degenerative disc diseases and, ultimately, ideally achieving IVD regeneration.

Evidence collected from various experimental models demonstrates that the cell’s response to a change in the microenvironment highlights an adaptive crosstalk between the molecules, changes in their subcellular colocalization, or even a replacement of partner, with a possible consequent loss or change of function. In many cases, these dynamic events may be underestimated due to the speed of occurrence, especially in a context like that of the IVD, which is characterized by very low cellularity (Raj, 2008; Fearing et al., 2018). However, the development of increasingly sophisticated investigation techniques can make an important contribution, also paving the way to the discovery of unexpected roles of specific proteins in the complicated microenvironment of the IVD (Dou et al., 2021).

In the present paper, we demonstrated the close relationship between the NFATc1 transcription factor and P2X7R in IVD cells. As the degree of IVD degeneration increased, the expression levels of these two proteins also increased. Data from NFATc1 overexpression and ChIP experiments support our hypothesis that NFATc1 is a positive modulator of P2X7R expression. It is therefore reasonable to define both of these two proteins as novel markers associated with IDD. Moreover, we found that NFATc1 and P2X7R associate closely in protein complexes both in the cytoplasm and in the nucleus of IVD cells from patients with different grading of degeneration, suggesting new hints for the identification of novel potential partners of the P2X7R.

The functional consequences of the interaction between NFATc1 and P2X7R deserve to be investigated in several directions as this will certainly help better understand P2X7R involvement in diverse sets of cellular responses. This is in agreement with data collected in recent years and coming from different experimental models which allow us to realize that neither the activity of the ligand-bound ion channel nor the permeability to large molecules completely explain all the roles/functions of P2X7R, since this receptor can interact directly with proteins participating to different intracellular signaling pathways (Kopp et al., 2019; Zumerle et al., 2019). Interactions between P2X7R and intracellular signal transduction proteins (protein kinases, phosphatases, and phospholipases), epithelial membrane proteins, or NLRP inflammasome multi-protein complexes have been proposed in different cellular models (Orioli et al., 2017). However, we are still far from a comprehensive understanding of the actions of P2X7R and the effects of its blockade as a therapeutic option. The existence of numerous conflicting reports and contrary conclusions on P2X7R’s roles in different fields (including inflammation, infections, energy metabolism, bone and cartilage metabolism, cancer metabolism, neurodegeneration, neurotransmitter release, cognition, and the development of the nervous system) is the evidence that P2X7R exerts pleiotropic effects on a specific cell population and that it can act as a multidirectional regulator depending on the degree of the stimulus. Searching for evidence to support P2X7R as a mechanistic biomarker, i.e., deciphering its specific role during physiological and pathologic conditions, can be much more informative than considering it as one of many descriptive biomarkers that often result as a side product of the disease. Unfortunately, the contribution of P2X7R to specific signaling pathways during normal physiology and pathology, as well as the complex regulatory network controlling P2X7R expression and function, has only been partially revealed. About the evidence we have obtained on the close relationship between P2X7R and NFATc1, which is a regulator of *P2RX7* gene promoter activity, it is worth asking questions: can P2X7R act by sequestering one of its transcriptional activators or repressors in a specific protein complex, with the aim of modulating its regulatory activity? In other words, does P2X7R participate in regulating its expression levels according to the needs of the cells? Hence, our interest in understanding the adaptive response of IVD cells led us to investigate the effect of the hypoxic environment on the P2X7R expression levels. We showed that under hypoxia-mimicking conditions, i.e., in CoCl_2_-treated cells, accompanied by an increase in HIF-1α, P2X7R expression levels significantly decreased. Interestingly, under these conditions, there is also increased recruitment of HIF-1α to the *P2RX7* promoter along with a decrease in NFATc1 occupancy, resulting in decreased P2X7R expression. This suggests that a decrease in P2X7R expression levels can be achieved by mimicking *in vitro* the hypoxic situation experienced by healthy IVD cells *in vivo* (Kerr et al., 2017; Kim et al., 2021b). This might be one of those conditions/strategies that favor the restoration of a functional IVD microenvironment.

Another scenario that certainly deserves to be explored is the interaction between lamin A/C and P2X7R. We are well aware of the technical hurdles limiting the experimental design; however, the observations we have collected allow the formulation of several hypotheses:

- P2X7R may contribute to the stability of the nucleus and play a role in gene expression regulation by participating in a signalosome with lamin A/C (Makarov et al., 2019) or other nuclear membrane proteins (Wong et al., 2022).
- Alternatively, P2X7R may act as a scaffold protein for intermediate filament proteins forming the inner layer of the nuclear envelope (the nuclear lamina) (Gruenbaum and Foisner, 2015) and affect nuclear physiological events, including chromatin remodeling and mechanotransduction (Manda et al., 2023).
- Accumulation of progerin, a truncated unprocessed lamin A protein causing systemic laminopathies (Cenni et al., 2020), was found in the pathogenesis of IDD, but its role is unclear (Xu et al., 2019). A possible P2X7R/lamin A/C crosstalk would aid in the identification of novel therapeutic targets and pharmacological regimens preventing or alleviating IDD.

Finally, whether P2X7R can promote both a non-genomic and genomic response to mechanical stimuli in IVD cells via a signalosome with NFATc1 or lamin A/C and thus participate in the physiological and pathological homeostasis of the human IVD remains a crucial investigation that may have clinical significance for innovative therapies for patients suffering from IDD.

Therefore, we believe that findings on these topics will substantially improve our understanding of the role of P2X7R in different pathological conditions and open an entirely new scenario on the pathophysiological functions of the P2X7R.

The use of the human experimental model, which inevitably cannot provide an adequate number of cells from patient biopsies, requires proceeding step by step. Therefore, the elucidation of the functional consequences of the interaction between NFATc1 and P2X7R or between lamin A/C and P2X7R will be the aim of the next investigations.

## Data Availability

The raw data supporting the conclusion of this article will be made available by the authors, without undue reservation.
