# Supplementary figures and images for: The NFATc1/P2X7 receptor relationship in human intervertebral disc cells
Source: Front Cell Dev Biol. 2024 Apr 4;12:1368318. doi: 10.3389/fcell.2024.1368318 (PMC11024252; doi:10.3389/fcell.2024.1368318)

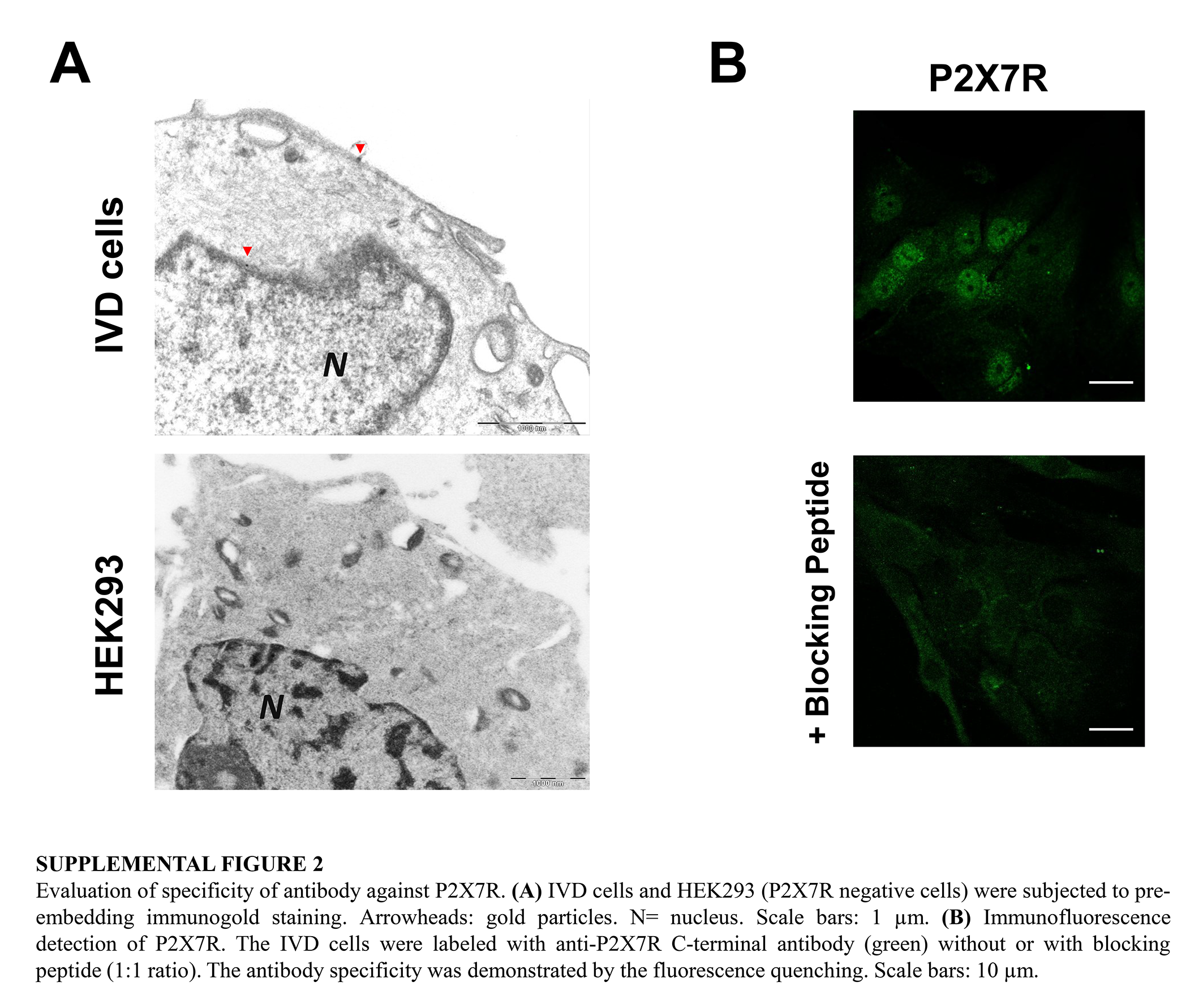

Supplement: Supplementary file 1 [file Image2.TIF]

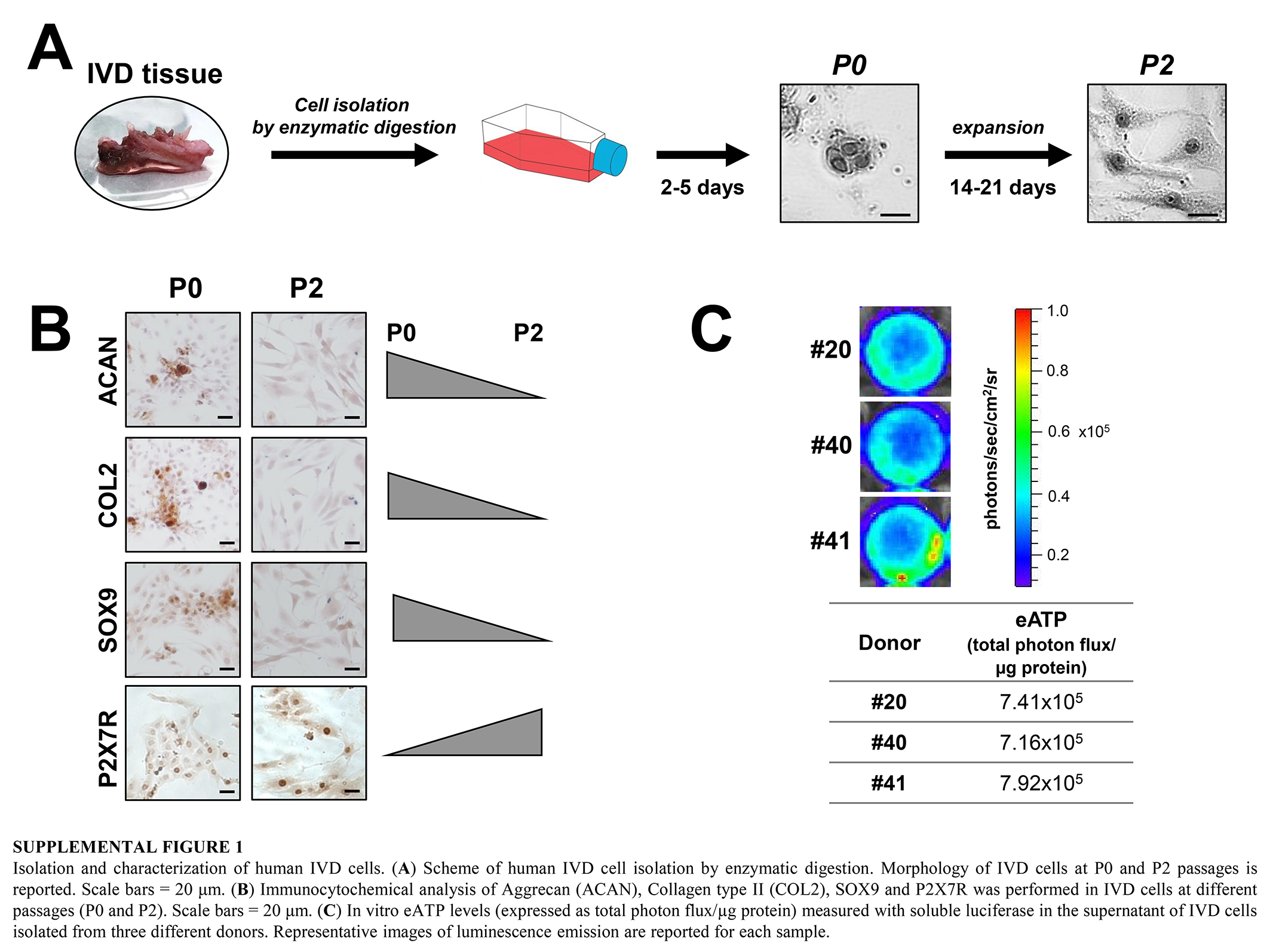

Supplement: Supplementary file 2 [file Image1.TIF]
